# Clinical value of serum cholinesterase levels in Nephrotic syndrome: an observational study

**DOI:** 10.1186/s12882-022-02764-0

**Published:** 2022-04-02

**Authors:** Kimihiko Goto, Keiji Kono, Hideki Fujii, Shunsuke Goto, Shinichi Nishi

**Affiliations:** 1 Division of Nephrology and Kidney Center, Kobe University Graduate School of Medicine, 7-5-2, Kusunoki-cho, Chuo-ku, Kobe, Hyogo 650-0017 Japan; 2 Division of Nephrology, Akashi medical Center, 743-33, Yagi, Ohkubo-cho, Akashi, Hyogo 674-0063 Japan

**Keywords:** Nephrotic syndrome, Cholinesterase, Minimal change nephrotic syndrome

## Abstract

**Background:**

Nephrotic syndrome (NS) results in massive proteinuria and hypoalbuminemia, which are responsible for a compensatory increase in protein synthesis in the liver. Serum cholinesterase (ChE) also increases in NS. However, its clinical value is not fully elucidated.

**Methods:**

In this study, 184 patients with NS who underwent kidney biopsy were included. The patients were divided into two groups according to serum ChE levels, as follows: hypercholinesterasemia (HC) and non-hypercholinesterasemia (NHC) groups. The clinical factors were compared between the two groups.

**Results:**

The HC group had significantly more severe proteinuria and higher prevalence of high selective proteinuria than the NHC group. Furthermore, the prevalence of minimal change nephrotic syndrome (MCNS) was significantly higher in the HC group than that in the NHC group. Multivariate analysis revealed that the severity of proteinuria and MCNS were significantly associated with HC.

**Conclusion:**

In this study, HC in NS was associated with the severity of proteinuria and MCNS, and could help clinicians predict the histological diagnosis of NS.

**Supplementary Information:**

The online version contains supplementary material available at 10.1186/s12882-022-02764-0.

## Introduction

Nephrotic syndrome (NS) is a glomerular disease characterized by severe proteinuria, hypoalbuminemia, and edema [1, 2]. NS can cause various complications, such as dyslipidemia [3, 4] and coagulation disorders [4–6]. These complications can be mostly explained by the compensatory increase in protein synthesis in the liver in response to a loss of various proteins, including albumin (Alb) into the urine [7, 8], although the details of its pathophysiology remain uncertain.

Cholinesterase (ChE) is one of the enzymes produced in the liver and belongs to the family of serine hydrolases [9]. Clinically, ChE is widely measured as a valuable marker for evaluating liver function, particularly protein synthesis [10, 11]. In contrast, this enzyme can also increase in NS. A study has reported that almost all patients with NS had hypercholinesterasemia (HC) [12]. However, the evidence of HC in NS has not been fully elucidated.

To resolve this issue, we investigated the clinical value of HC in NS.

## Methods

### Study enrollment

This study adopted a single-center retrospective observational study design, and adult patients with new-onset NS who underwent kidney biopsy at our hospital from April 2010 to March 2019 were included. We excluded patients under the age of 18 years, those with insufficient data on serum ChE, and those with decompensated liver cirrhosis. Finally, 184 subjects were included in this study. Our protocols were approved by the Kobe University Clinical Research Ethical Committee (no. B190327) and performed according to the recommendations of the Declaration of Helsinki for Biomedical Research involving human subjects. The Kobe University Clinical Research Ethical Committee approved to waive off the need for an informed consent and to use the opt-out approach in the study, because the data were retrospectively and anonymously analyzed.

### Data collection and definitions

All clinical data were collected from the patients’ medical records. Urine and blood samples, including ChE, were measured using standard laboratory techniques (JCA-BM8040G BioMajesty™, JEOL, Tokyo, Japan). Serum ChE activity was determined using an assay with p-hydroxybenzoylcholine iodine salt as the substrate (Shino-Test Corporation, Kanagawa, Japan). HC was defined as elevated ChE levels above the upper limit of normal reference ranges in our hospital (male: 240–486 U/L; female: 201–421 U/L). All subjects were divided into two groups, as follows: the HC and non-HC (NHC) groups. Subsequently, we compared clinical characteristics between the two groups. Furthermore, in order to investigate the association of HC with proteinuria and histopathological diagnosis, all the patients were divided into six groups according to the tertiles of proteinuria levels (T1:lowest, T3:highest) and the histopathological diagnosis (MCNS and non-MCNS), and we compared the prevalence of HC in each category.

The histopathological diagnosis of kidney biopsy was determined in our pathology department. NS was defined as proteinuria ≥3.5 g/day or ≥ 3.5 g/g Cre and serum Alb ≤3.0 g/dL, according to the guidelines of the Japanese Society of Nephrology [1]. As for the selectivity of proteinuria, we calculated the selectivity index (SI) as follows [13]: SI = (urine immunoglobulin (Ig) G/serum IgG) × (serum transferrin/urine transferrin). High selective proteinuria was defined as SI ≤ 0.2 according to a previous report [13]. Hypercholesterolemia was defined as total cholesterol (T-chol) ≥ 220 mg/dL.

### Statistical analysis

Data are expressed as mean ± standard deviation, median and interquartile range, or proportions. Differences between the two groups were tested using the unpaired t-test, Mann–Whitney U-test, and chi-square test, as appropriate. Furthermore, we conducted a logistic regression analysis for HC. *P*-values of less than 0.05 were used to denote statistical significance. All statistical analyses were performed using Statistical Package for the Social Sciences, version 25.0 (IBM Corp., Armonk, NY, USA).

## Results

### Patient characteristics

Table 1 shows the characteristics of the patients enrolled in this study. Among the 184 patients, 72 (39.1%) had HC. The proportion of male patients, those with diabetes, those with liver diseases including hepatitis and cirrhosis, and those who use statins was similar between the two groups. In contrast, the patients in the HC group were significantly younger, had better kidney function, had lower blood pressure and serum Alb levels, and had higher total cholesterol (T-Chol) levels than those in the NHC group. The median serum ChE level was 565 U/L in the HC group and 355 U/L in the NHC group.

**Table 1:** Baseline characteristics of patients in each group

	HC (***N*** = 72)	NHC (***N*** = 112)	***P*** value
Age (years old)	51 (33–68)	66 (46–74)	< 0.05
Sex (male) (*n* (%))	35 (48.6)	58 (51.8)	NS
DM (*n* (%))	8 (11.1)	15 (13.4)	NS
Liver diseases (n(%))	5 (6.9)	14 (12.5)	NS
Statin (*n* (%))	15 (20.8)	37 (33.0)	NS
Mean BP (mmHg)	88 ± 12	93 ± 14	< 0.05
Serum ChE (U/L)	565 (509–643)	355 (270–401)	< 0.05
Serum Cre (mg/dL)	0.83 (0.65–0.99)	1.01 (0.71–1.59)	< 0.05
eGFR (mL/min/1.73 m^2^)	69.3 (54.5–84.0)	52.8 (31.0–72.7)	< 0.05
Alb (g/dL)	1.6 (1.2–2.2)	2.4 (1.7–2.8)	< 0.05
T-chol (mg/dL)	430 (352–531)	281 (224–340)	< 0.05
Proteinuria (g/day)	6.4 (4.5–9.4)	4.3 (3.5–7.3)	< 0.05
Selectivity index ≤ 0.2 (n(%))	54/67 (80.6)	38/92 (41.3)	< 0.05
MCNS (n(%))	42 (58.3)	13 (11.6)	< 0.05

Values are expressed as mean ± standard deviation or median and interquartile or proportion

*Abbreviations*; *HC* hypercholinesterasemia, *NHC* non-hypercholinesterasemia, *DM* diabetes mellitus, *BP* blood pressure, *ChE* cholinesterase, *Cre* creatinine, *eGFR* estimated glomerular filtration rate, *Alb* albumin, *T-chol* Total cholesterol, *MCNS* minimal change nephrotic syndrome, *NS* not significant

### Comparison of proteinuria and histopathological diagnosis between the two groups

**Fig. 1:**
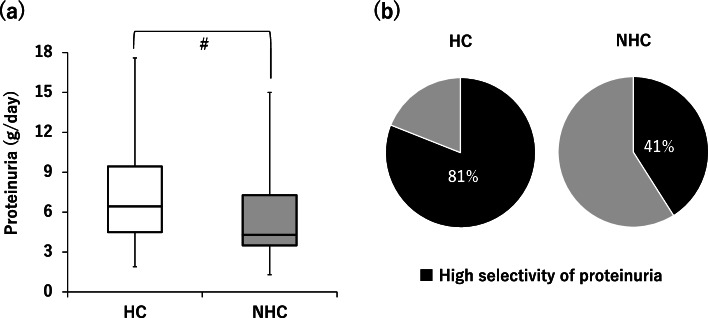
Comparison of clinical data on proteinuria between the two groups. **a**: The amount of proteinuria. **b**: The proportion of patients with high selective proteinuria (selectivity index of proteinuria ≤0.2). Abbreviations; HC: hyper-cholinesterasemia, NHC: non hyper-cholinesterasemia

**Fig. 2:**
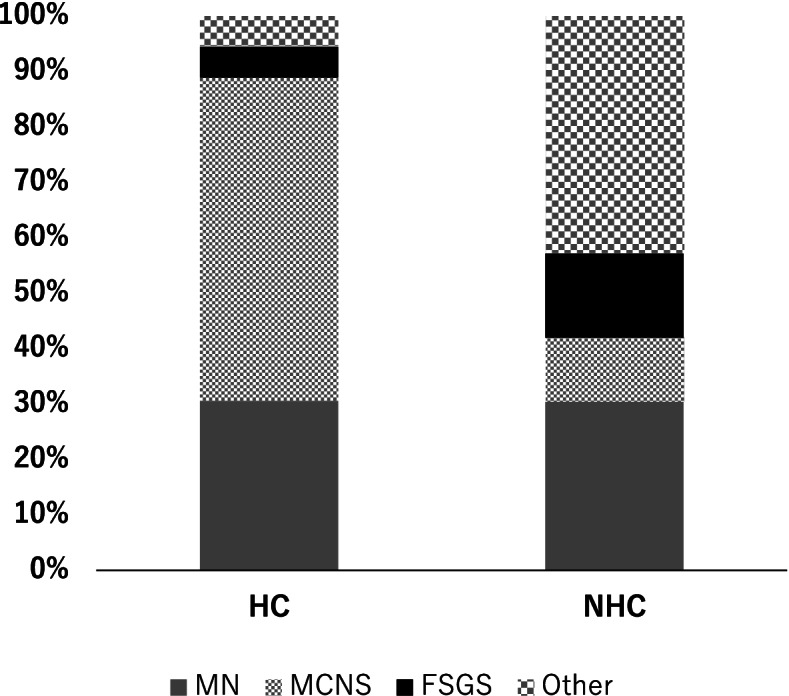
Comparison of histopathological diagnosis of NS between the two groups. Abbreviations; NS: nephrotic syndrome, HC: hyper-cholinesterasemia, NHC: non hyper-cholinesterasemia, FSGS: focal segmental glomerulosclerosis, MCNS: minimal change nephrotic syndrome, MN: membranous nephropathy

The HC group had significantly more severe proteinuria (HC: 6.4 g/day (range, 4.5–9.4 g/day); NHC: 4.3 g/day (range, 3.5–7.3)) (*p* <  0.05) and higher prevalence of high selective proteinuria than the NHC group (Fig. 1). As for the histopathological diagnosis, the HC group had a significantly higher prevalence of minimal change nephrotic syndrome (MCNS) than the NHC group (Fig. 2).

### Clinical factors associated with HC

Univariate logistic regression showed that HC was significantly associated with the following factors: age, mean blood pressure, estimated glomerular filtration rate, serum Alb, T-Chol, proteinuria severity, high selective proteinuria, and MCNS (Table 2). After adjusting for potential confounders, HC was significantly associated with proteinuria severity (adjusted odds ratio: 1.21 (range, 1.04–1.40)) (*p* <  0.05) and MCNS (adjusted odds ratio: 4.66 (range, 1.39–15.64)) (*p* <  0.05).

**Table 2:** Factors affecting HC in subjects with NS

(a) univariate logistic analyses of the variables for HC						
		Odds ratio	95% CI		*P* value	
Age (years)		0.972	0.957–0.989		< 0.05	
Mean BP		0.968	0.945–0.992		< 0.05	
eGFR (mL/min/1.73 m^2^)		1.024	1.011–1.036		< 0.05	
Alb (g/dL)		0.304	0.184–0.502		< 0.05	
Proteinuria (g/day)		1.142	1.044–1.249		< 0.05	
T-chol (mg/dL)		1.011	1.008–1.015		< 0.05	
Selectivity Index ≤ 0.2		5.903	2.833–12.299		< 0.05	
MCNS		10.662	5.066–22.440		< 0.05	
(b) multivariate logistic analyses of the variables for HC						
	Model-1			Model-2		
	Odds ratio	95% CI	*P* value	Odds ratio	95% CI	*P* value
Proteinuria (g/day)	1.172	1.022–1.344	< 0.05	1.210	1.044–1.403	< 0.05
MCNS	5.874	2.064–16.715	< 0.05	4.660	1.389–15.635	< 0.05

Model 1: adjusted for age and mean BP, eGFR, Alb, T-chol; Model 2: further adjusted for the presence of high selectivity of proteinuria

*Abbreviations*; *HC* hyper-cholinesterasemia, *BP* blood pressure, *eGFR* estimated glomerular filtration rate, *Alb* albumin, *T-chol* Total-cholesterol, *MCNS* minimal change nephrotic syndrome

### Association between HC and proteinuria severity and histopathological diagnosis

**Fig. 3:**
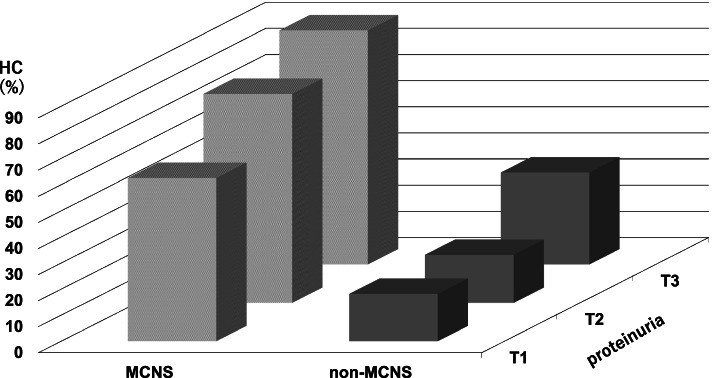
Relationship between the prevalence of HC, the amount of proteinuria and histopathological diagnosis. Abbreviations; HC: hyper-cholinesterasemia, MCNS: minimal change nephrotic syndrome. T: tertile

To clarify the association of HC with proteinuria and histopathological diagnosis, the patients were divided into six groups according to the tertiles of proteinuria levels and histopathological diagnosis (MCNS and non-MCNS), and we compared the prevalence of HC in each category. The prevalence of HC increased along with more severe proteinuria in both the MCNS and non-MCNS groups, and the prevalence was significantly higher in the MCNS group than that in the non-MCNS groups, across all tertiles of proteinuria (Fig. 3). Interestingly, the prevalence of HC was higher in MCNS T1 compared to non-MCNS T3, although serum Alb levels were comparable between non-MCNS T3 and MCNS T1 (median serum Alb levels; non-MCNS T3: 2.0 g/dL, MCNS T1: 1.8 g/dL).

## Discussion

In this study, we demonstrated the following results: (1) the incidence of HC was approximately 40% in patients with NS, (2) the HC group had significantly more severe proteinuria and higher prevalence of high selective proteinuria than the NHC group, (3) the prevalence of MCNS was significantly higher in the HC group than that in the NHC group, (4) multivariate analysis revealed that HC was significantly associated with proteinuria severity and MCNS, and the prevalence of HC was significantly higher in the MCNS group than that in the non-MCNS group, irrespective of proteinuria severity.

NS can increase serum ChE levels, which is based on the results of previous studies [12, 14]. Way et al. have reported that serum ChE levels were higher in the NS group (*N* = 16) than those in the healthy group [14]. Furthermore, Vorhaus LJ et al. have reported that 95% of subjects with NS (*N* = 19) had HC [12]. However, in this study, the incidence of HC in patients with NS was approximately 40%, which was lower than those reported in two previous studies. Although our results are inconsistent with theirs, their studies differed from ours in several points. In the previous studies, the sample size was relatively small, and the information on the patients’ clinical characteristics was insufficient. Considering these facts, it is considered that much remains unknown regarding the clinical value of serum ChE in patients with NS. Therefore, we conducted more detailed examination on the clinical characteristics of HC in a larger number of patients with NS.

First, the HC group had significantly more severe proteinuria than the NHC group. The hepatic synthesis of various proteins, including Alb and ChE, is enhanced in response to the loss of Alb from the kidney among patients with NS [7, 8, 12]. As the mechanisms of enhanced protein synthesis, it is thought that a decline in colloid osmotic pressure, caused by a decrease in the concentration of various proteins, triggers protein synthesis in the liver [15]. In fact, it has been reported that the amount of loss of Alb into the urine exhibits a positive correlation with the amount of synthesis of Alb in the liver [7, 16]. Furthermore, a study has reported that ChE synthesis occurred in parallel with Alb synthesis [12]. Considering these results, we speculated that HC was related to proteinuria severity in patients with NS. In contrast, multivariate analysis did not show that HC was related to serum Alb levels in this study. The reason could be partly explained by the difference in the amount of protein loss into the urine depending on the types of protein. A study has reported that ChE was scarcely lost into the urine in patients with NS [12], while Alb is a chief component of urine protein. Since serum Alb levels in NS can be partly determined by the balance between Alb loss from the kidneys and Alb synthesis from the liver, the absolute level of serum Alb may fail to fully reflect Alb included in the urine alone. Conversely, ChE is scarcely lost into the urine, and therefore, serum ChE levels in NS can easily reflect the enhanced ChE synthesis in the liver, which is derived from Alb loss from the kidneys. From these findings, we assumed that HC was significantly related to the severity of proteinuria, rather than serum Alb levels.

Second, our results demonstrated that the HC group had a significantly higher prevalence of high selective proteinuria than the NHC group. The results of a study have indicated that patients with highly selective proteinuria had a more intensive loss of Alb into the urine than those with less selective proteinuria, even if both have the same proteinuria severity [13]. As stated above, the increase in serum ChE levels in patients with NS results from the homeostatic mechanism against protein loss, especially Alb. Therefore, we assumed that one of the necessary conditions leading to HC in NS might be severe proteinuria and highly selective proteinuria, resulting in a significantly larger amount of Alb leakage into the urine.

**Fig. 4:**
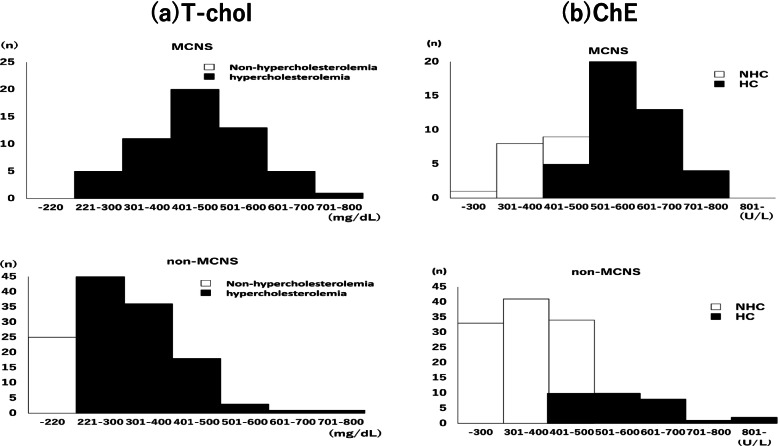
Distribution of (**a**) serum T-chol and (**b**) serum ChE levels. **a** Distribution of serum T-chol levels. White bar; Non-hypercholesteremia, Black bar; hypercholesteremia. **b** Distribution of serum ChE levels. White bar; NHC, Black bar; HC. Abbreviations; T-chol: Total-cholesterol, ChE: cholinesterase, HC: hyper-cholinesterasemia, NHC: non hyper-cholinesterasemia, MCNS: minimal change nephrotic syndrome

Third, histopathologically, the proportion of patients with MCNS was significantly larger in the HC group. We attempted to explain this result based on its clinical characteristics. The characteristics of MCNS include highly selective proteinuria [13, 17] and rapid onset [18–20]. In fact, the selectivity of proteinuria was significantly better in the MCNS group than that in the non-MCNS group in this study (SI: 0.12 vs. 0.26, respectively). Moreover, the MCNS group had a significantly higher prevalence of HC in all tertiles of proteinuria levels than the non-MCNS group. These results suggested that MCNS causes intensive leakage of Alb into the urine, which resulted in a compensatory and drastic increase in protein synthesis in the liver, because of highly selective proteinuria. Indeed, patients with MCNS had higher ChE and T-chol levels simultaneously than the non-MCNS group (Fig. 4). As for the rapid onset, no studies have examined the association of ChE synthesis with the rate of decline in serum Alb levels. As Fig. 3 shows, the prevalence of HC was higher in MCNS T1 compared to non-MCNS T3 although serum Alb levels were similar between MCNS T1 and non-MCNS T3. This result suggested that there was a possibility that HC could be influenced not only by the severity of hypoalbuminemia and proteinuria, but also by the other factor. Although this study also lacked information on the time course of the onset of NS, we speculated that the rapid decline in serum Alb could contribute to the stimulation of ChE synthesis. Further study is needed to resolve this issue.

In daily clinical practice, the histopathological diagnosis of NS is critical information for determining the indication of treatment and subsequent prognosis. The standard method for the histopathological diagnosis is a kidney biopsy. Since it is an invasive procedure and cannot be routinely performed, an alternative diagnostic approach is also important. The histopathological diagnosis is predicted based on clinical information, such as the onset of NS and the selectivity of proteinuria; however, the SI of proteinuria may not always be easily obtained. In contrast, ChE measurement is simple and quick and can be performed in many institutions. This study demonstrated the relationship between HC and MCNS, and therefore, we considered that ChE facilitates the prediction of NS tissue types in clinical settings. T-chol also can be easily measured, and the absolute value of both T-chol and ChE was significantly higher in MCNS compared to non-MCNS in this study. However, Fig. 4 showed that almost all patients had hypercholesterolemia in both MCNS and non-MCNS (MCNS 100%, non-MCNS 81%), while the prevalence of HC was different between the two groups (MCNS 76%, non-MCNS 23%). This result suggested that HC was more useful for predicting the histological diagnosis of NS compared to hypercholesterolemia.

This study has some limitations. (1) The sample size was small, and the logistic analysis had limitations. However, we believe that this study is valuable since only a few studies have investigated the clinical value of ChE in patients with NS. (2) There were no data on ChE before the onset of NS because of the retrospective observational nature of this study. (3) The number of patients with NS other than MCNS and membranous nephropathy was relatively small. To overcome these limitations, further examination is required.

In conclusion, our results suggested that HC was associated with severe proteinuria and MCNS in NS and could help clinicians predict the histological diagnosis of NS.

## Supplementary Information

**Additional file 1.**

## Data Availability

All data generated or analyzed during this study are included in this published article and its supplementary information files.

## Abbreviations

NS: Nephrotic syndrome
Alb: Albumin
ChE: Cholinesterase
HC: Hypercholinesterasemia
NHC: Non-hypercholinesterasemia
MCNS: Minimal change nephrotic syndrome
T-chol: Total-cholesterol
Ig: Immunoglobulin
